# Cryolipolysis in Aesthetic Plastic Surgery

**Published:** 2014-08-22

**Authors:** Karan Chopra, Kashyap K. Tadisina, W. Grant Stevens

**Affiliations:** ^a^Department of Plastic and Reconstructive Surgery, Johns Hopkins School of Medicine, Baltimore, Md; ^b^Division of Plastic Surgery, University of Maryland Medical Center, Baltimore, Md; ^c^University of Illinois at Chicago College of Medicine, Chicago, Ill; ^d^Marina Plastic Surgery Associates and Sientra, Inc, Marina del Rey and Santa Barbara, Calif

**Keywords:** cryolipolysis, body sculpting, nonsurgical fat reduction, noninvasive body contouring, Coolsculpting

**Figure:**
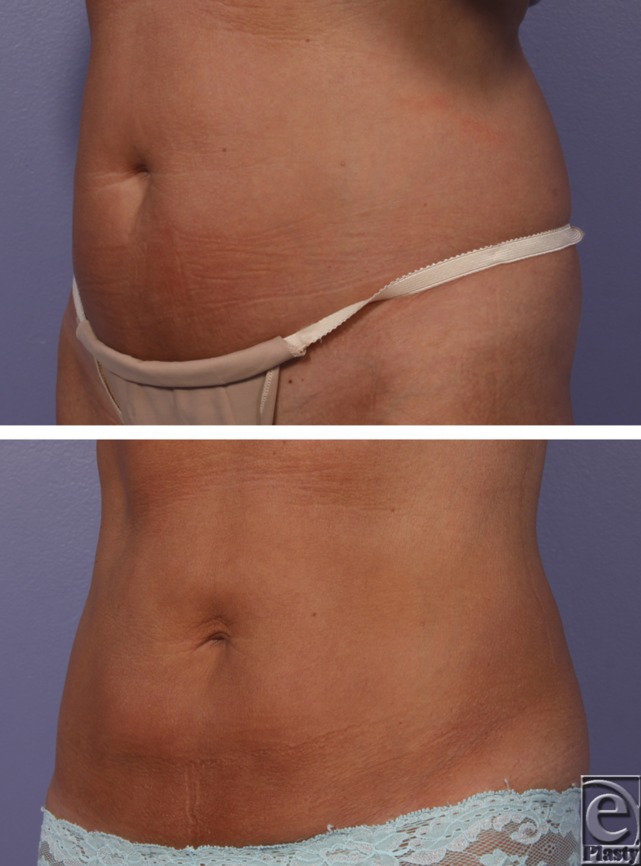


## DESCRIPTION

A pleasant 50-year-old woman presented to the plastic surgery clinic with complaints of persistent localized excess fat in her lower abdomen despite improved diet and exercise. Cryolipolysis of her abdomen was performed, and she returned to clinic for her 8-week follow-up.

## QUESTIONS

1. **What is cryolipolysis, and what is its role in plastic surgery?**
2. **What is the mechanism by which cryolipolysis works?**
3. **What are the recognized shortcomings and disadvantages of cryolipolysis?**
4. **How effective has cryolipolysis been shown to be and what is its future in aesthetic surgery?**

## DISCUSSION

Cryolipolysis is the result of continued innovation and development in the area of noninvasive body contouring in aesthetic plastic surgery.^1^^,^^2^ The demand for body contouring procedures is rising because of the advent of bariatric surgery. Body contouring procedures allow surgeons to treat isolated areas of excess fat that lead to asymmetric appearance and adiposity in unwanted locations.^2^ Currently, liposuction is the most frequently employed and effective technique for body contouring but, due to its invasive nature, comes with inherent risks including bleeding and infection with an associated longer recovery time. Furthermore, one of the most common complications of liposuction is contour irregularities. Other noninvasive body contouring methods that have been described with varying degrees of success include: infrared light, lasers, radiofrequency, massage, and even high-frequency ultrasound techniques,^3^ yet their long-term viability and consistency in results have not been proven. Cryolipolysis is a recent technology used for controlled, natural, and selective fat reduction utilizing localized cooling to extract heat from adipocytes.^1^ Although there are no established, formal indications of when to use this technology, it is used in a variety of clinical situations, from the patient who desires scarless reduction of adipose tissue to those who are unfit to tolerate the anesthesia required for more invasive forms of liposuction.

Although the exact mechanism is still being studied, cryolipolysis works at a cellular level by an overarching theme of inflammation followed by phagocytosis and apoptosis. The basic principles of cryobiology date back to the 1960s when research in rapid freezing and concurrent ischemia were being studied.^4^ Adipose tissue, as compared to other tissues, is more sensitive to cold temperatures.^4^ Studies performed in porcine and in vitro models, with histological and pathological studies, confirm this cell response. Adipose cells undergo an inflammatory response after exposure to cold temperatures (-1 to -7°C) within the first 72 hours, peaking at 14 days after treatment. Between 14 and 30 days, phagocytosis of adipose cells begins. By 60 to 90 days, the inflammatory process declines and the adipose cell volume decreases with concurrent interlobular septal thickening. This roughly 90-day cycle results in selective subcutaneous fat layer reduction.^1^

Although one of the greatest advantages to cryolipolysis is its safety in the population, there have been documented unwanted side effects that occur. Symptoms include erythema, edema, decreased sensation, and pain in the treatment area, but none have been reported as permanent.^5^ Biopsy studies of nerve fibers confirm this short-term decrease in sensation being temporary, with no permanent changes in nerve fibers, and sensation returning in patients by 3 to 4 weeks.^6^ One area warranting further study is the efficacy of cryolipolysis in patients with cold-induced medical conditions such as cryoglobulinemia, cold urticaria, and paroxysmal cold hemoglobinuria need further testing.^1^ Isolated case studies report incidences of delayed paradoxical hyperplasia at sites of cryolipolysis as 2 to 3 months,^7^ with unintended adipose tissue growth stimulation as the hypothesized mechanism. Finally, although found to be effective, cryolipolysis is not a suitable replacement for high-volume liposuction because traditional liposuction can remove more adipose tissue whereas cryolipolysis is not intended for high volume removal of adipose tissue in a single sitting.

Cryolipolysis has been found to be effective through porcine model studies as well as numerous recently published clinical studies. Porcine model studies found an 80% reduction in superficial fat layers and 40% total fat layer reduction at 3.5 months posttreatment.^8^ A volumetric quantification study found that per cycle, close to 40 cc of adipose tissue is lost in flank areas 2 months posttreatment.^5^ Clinical studies have shown cryolipolysis to be efficacious in reducing fat in the lower and upper abdomen, inner and outer thighs, flank area, and back.^2^ In terms of longevity, case studies report fat reduction sustained for 2 to 5 years posttreatment. Finally, studies show more than 80% satisfaction rate among patients,^5^ with more than 80% of patients willing to recommend treatment to a friend. Overall, cryolipolysis offers patients a safe, effective, controlled, and noninvasive method to deal with unwanted fat in various parts of the body.

## References

[1] Shek SY, Chan NP, Chan HH (2012). Non-invasive cryolipolysis for body contouring in Chinese—a first commercial experience. Lasers Surg Med.

[2] Stevens WG, Pietrzak LK, Spring MA (2013). Broad overview of a clinical and commercial experience with CoolSculpting. Aesthet Surg J.

[3] Tadisina KK, Patel MN, Chopra K (2013). High-intensity focused ultrasound in aesthetic plastic surgery. Eplasty.

[4] Sasaki GH, Abelev N, Tevez-Ortiz A (2014). Noninvasive selective cryolipolysis and reperfusion recovery for localized natural fat reduction and contouring. Aesthet Surg J.

[5] Garibyan L, Sipprell WH, Jalian HR, Sakamoto FH, Avram M, Anderson RR (2014). Three-dimensional volumetric quantification of fat loss following cryolipolysis. Lasers Surg Med.

[6] Coleman SR, Sachdeva K, Egbert BM, Preciado J, Allison J (2009). Clinical efficacy of noninvasive cryolipolysis and its effects on peripheral nerves. Aesthetic Plast Surg.

[7] Jalian HR, Avram MM, Garibyan L, Mihm MC, Anderson RR (2014). Paradoxical adipose hyperplasia after cryolipolysis. JAMA Dermatol.

[8] Manstein D, Laubach H, Waaranabe K, Farinelli W (2009). Selective cryolysis: a novel method of non-invasive fat removal. Lasers Surg Med.

